# EV-Mediated Chemoresistance in the Tumor Microenvironment: Is NF-κB a Player?

**DOI:** 10.3389/fonc.2022.933922

**Published:** 2022-06-22

**Authors:** Mauro Di Vito Nolfi, Davide Vecchiotti, Irene Flati, Daniela Verzella, Monica Di Padova, Edoardo Alesse, Daria Capece, Francesca Zazzeroni

**Affiliations:** Department of Biotechnological and Applied Clinical Sciences (DISCAB), University of L’Aquila, L’Aquila, Italy

**Keywords:** extracellular vesicles, NF-κB, drug resistance, tumor microenvironment, inflammasome

## Abstract

Drug resistance is a major impediment to patient survival and remains the primary cause of unsuccessful cancer therapy. Drug resistance occurs in many tumors and is frequently induced by chemotherapy which triggers a defensive response both in cancerous and cancer-associated cells that constitute the tumor microenvironment (TME). Cell to cell communication within the TME is often mediated by extracellular vesicles (EVs) which carry specific tumor-promoting factors able to activate survival pathways and immune escape mechanisms, thus sustaining tumor progression and therapy resistance. NF-κB has been recognized as a crucial player in this context. NF-κB activation is involved in EVs release and EVs, in turn, can trigger NF-κB pathway activation in specific contexts, based on secreting cytotype and their specific delivered cargo. In this review, we discuss the role of NF-κB/EVs interplay that sustain chemoresistance in the TME by focusing on the molecular mechanisms that underlie inflammation, EVs release, and acquired drug resistance.

## Introduction

Cancer chemotherapy resistance is the innate and/or acquired ability of cancer cells to escape the effects of chemotherapeutics and represents a great challenge in cancer therapy to improve clinical outcomes. The development of resistance occurs in many tumors and depends partially on genetic instability, heterogeneity, speedy mutation in the tumor cell, cytogenetic changes, and intra-neoplastic diversity (1, 2). The tumor microenvironment (TME) is also considered to be a factor for resistance development in many cancers, as chemotherapy frequently triggers a defensive response not only in cancerous cells but also in cancer-associated cells within the TME (3). Activation of survival pathways and immune escape mechanisms are often mediate by extracellular vesicles (EVs), which release their cargo in recipient cells within TME. EVs are lipid-contained vesicle, which are classified based on their size, biogenesis, and release mechanism. EVs include microvesicles (MVs), exosomes (EXs) and apoptotic bodies (ABs) (4). MVs are 100 to 1000 nm in size, EXs are smaller and range in size from 30 to 150 nm, while ABs show a size ranging from 50 to 5000 nm. MVs and ABs directly bud from cytoplasmic membrane, while EXs are produced by inward budding of plasma membrane and formation of endosomes, which mature into multivesicular bodies (MVBs) and subsequently were secreted into extracellular space (5, 6). Among EVs, exosomes are the best characterized. After formation, early endosome is processed in MVBs by the endosomal sorting complexes required for transport (ESCRT), directing by the invagination of MVBs outer membrane and packaging of biomolecules (7). EVs support intracellular communications within the TME by carrying specific tumor-promoting factors that positively regulate several pro-survival pathways including NF-κB, which plays a pivotal role in this context.

NF-κB transcription factor family consists of a group of inducible effectors which regulate a plethora of genes involved in several physiological processes such as inflammation (8), differentiation (9), survival (10), proliferation (11), and immunity (12). However, dysregulated NF-κB signaling is often found in many pathological conditions including inflammatory disorders (13), autoimmunity (14), and cancer (15). NF-κB family comprises five structurally related members, specifically, p65 (RelA), RelB, c-Rel, p50 (NF-κB1), and p52 (NF-κB2) that form homodimers and heterodimers able to specifically bind the consensus κB site 5’-GGGPuNNPyPyCC-3 (16) with different transcriptional activity (17). p50 and p52 are active forms of precursors proteins p105 and p100 respectively that, in turn, operate both as NF-κB precursors and inhibitors of NF-κB dimers (18). Activation of NF-κB pathway occurs through two major mechanisms, namely canonical (or classical) and non-canonical (or alternative) pathways (19). In absence of stimuli, NF-κB dimers are retained within the cytosol in inactive forms through their interaction with IκB (inhibitor of kappa B) proteins (20). In the canonical pathway, upon stimulation [*i.e.*, lipopolysaccharide (LPS), tumor necrosis factor α (TNF-α)], IKK (IκB kinase) (21) proteins (IKKα, IKKβ, IKKγ) assemble into multiproteic complexes and trigger NF-κB activation. Specifically, in presence of stimuli, activated IKK complex phosphorylates IκB proteins, thus inducing its ubiquitination (22) and proteasomal degradation and allowing, in turn, NF-κB dimers to translocate into the nucleus and activate its target genes. In the non-canonical pathway, induced by a subset of tumor necrosis factor receptor (TNFR) superfamily members upon stimulation by several factors such as lymphotoxin B and BAFF, the activation of the heterodimers p100/RelB is triggered by NF-κB inducing kinase (NIK) that activates IKKα complex and in turn promote the processing of p100 in p52 and the nuclear translocation of the active p52/RelB dimer.

While the role of NF-κB in promoting cancer progression and drug resistance is well-known, emerging evidence are pointing out for a crucial interplay between NF-κB and EVs that sustain TME remodeling, tumor inflammation and therapy resistance. Here, we discuss this crosstalk by focusing on the molecular mechanisms that underlie inflammation, EVs release, and acquired drug resistance.

### NF-κB: A Master Regulator of Inflammation and Therapy Resistance in Cancer

#### NF-κB Signaling in Inflammation and Cancer

During carcinogenesis, parenchymal cells continuously interact with the surrounding environment establishing a plethora of interactions with stromal cells (*i.e.*, fibroblasts, endothelial cells, adipose cells, mesenchymal stem cells), immune cells, and extracellular matrix that constitute the TME (23). In this context, inflammation plays a key role in contributing to carcinogenesis and promoting the metastatic phenotype (24, 25). NF-κB constitutive activation is widely recognized as a hallmark of many types of tumors including hepatocellular carcinoma (26), breast cancer (27), lymphoid malignancies (28), colorectal (29) and prostate cancer (30). In addition to promoting tumor cell survival, oncogenic NF-κB signaling operates in the TME, thereby linking inflammation and cancer (15, 31). The inflammatory response is mainly induced by TNF-α, Interleukin-1β (IL-1β), and Interleukin-6 (IL-6). These cytokines usually are not overexpressed in healthy tissues but are significant upregulated in response to several pathological stimuli. Although released to protect the host, these cytokines often trigger a positive feedback mechanism promoting a chronic inflammation that, in turn, sustains, carcinogenesis and tumor progression (32). Accordingly, NF-κB activation in non-malignant tumor-associated cells has been shown to amplify the production of cytokines and other specialized effectors that promote tumor-cell proliferation, invasion and therapy resistance, while suppressing anti-tumor immune responses (33). Damage-associated molecular patterns (DAMPs), are endogenous molecules produced by dying cancer cells in response to stress and cell injury (34). After production, DAMPs are secreted through several mechanisms including extracellular vesicles (EVs) (35); these are recognized by specific pattern recognition receptors (PRRs) expressed on several cells, such as monocytes and macrophages, which activate different inflammatory pathways including NF-κB pathway. In turn, NF-κB activation causes the release of proinflammatory cytokines, such as IL-1β (36). As part of a positive feedback loop, active IL-1β binds to IL-1 receptors (IL-1R) on cancer cells and further stimulate NF-κB signaling, thus inducing the expression of pro-inflammatory cytokines TNF-α and IL-6 and sustaining NF-κB-mediated chronic inflammation (37–39). Moreover, enhanced or deregulated NF-κB activity in fibroblasts and macrophages promotes their switching to cancer-associated fibroblasts (CAFs) and tumor-associated macrophages (TAMs), respectively, thus supporting tumor progression, vascularization and tumor growth, as observed in several cancers (40–45). NF-κB signaling also plays an important role in macrophages polarization (46); M1-type macrophages have a pro-inflammatory activity and tissue damaging properties, while M2 macrophages, with their anti-inflammatory phenotype, promote cell proliferation and tissue repair. Interestingly, although NF-κB represents a key transcription factor in M1 macrophage during the early stage of tumorigenesis, it also plays a pivotal role in advanced stages where it polarizes TAMs toward the immunosuppressive and tumor-promoting M2 phenotype. Indeed, we have demonstrated that NF-κB activation, through its target gene *GADD45B*, prevents TAM polarization to M1, thus inhibiting their antitumor activity (47, 48). Furthermore, it was observed that NF-κB p50 protein suppresses M1-type polarization and supports M2 immunosuppressive phenotype (49). The role of NF-κB in CAFs and TAMs polarization represents a hallmark of inflammatory TME and has been well explained in many excellent reviews (50, 51). In summary, as discussed below, NF-κB-mediated inflammation constitutes an important link between EVs activity and acquired drug resistance.

#### NF-κB-Driven Drug Resistance

**Figure 1:**
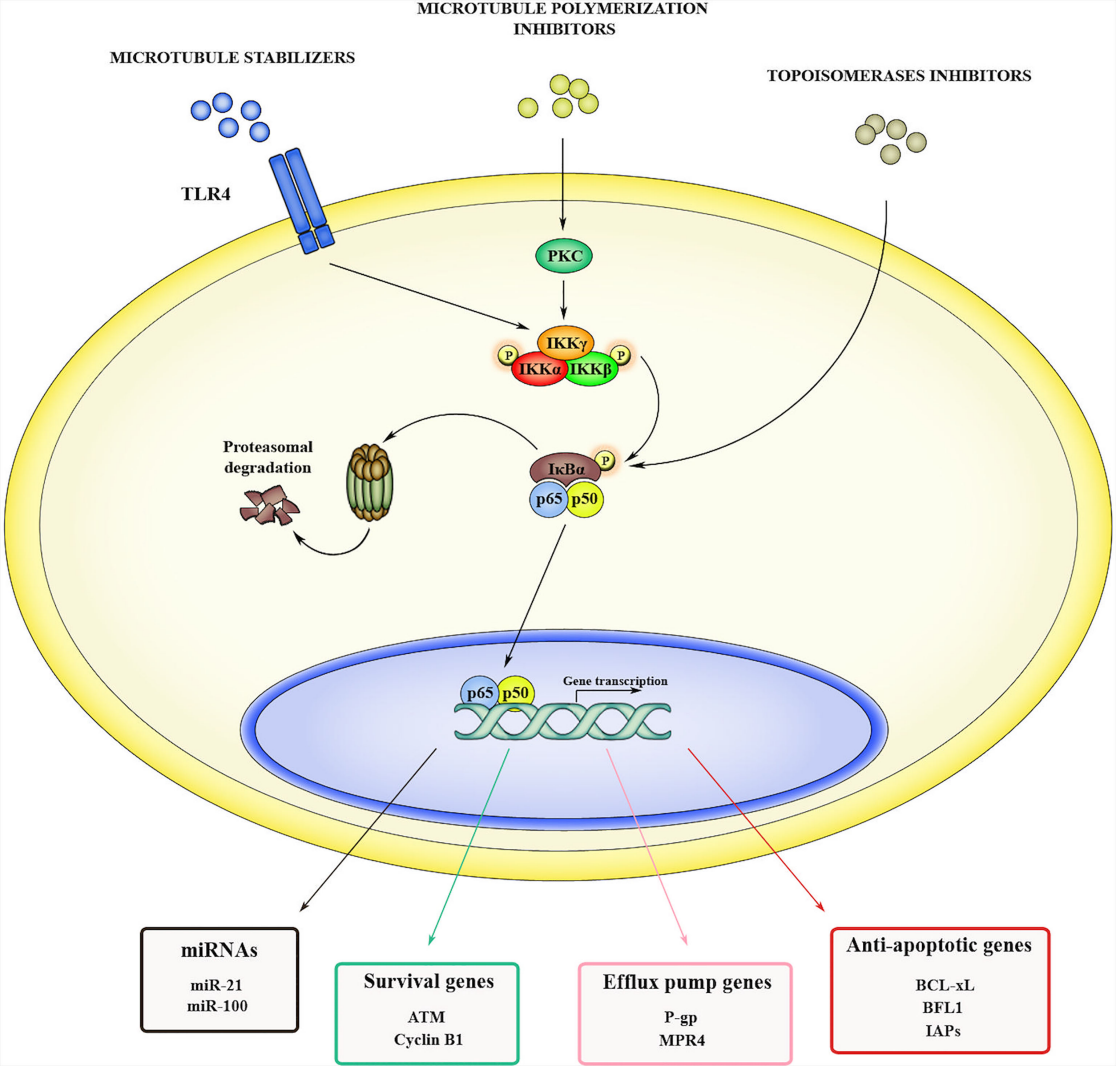
Mechanisms of NF-κB-induced drug resistance. NF-κB can be activated in cancer cells in response to several anticancer drugs, including microtubule stabilizers (*i.e.*, paclitaxel), microtubule polymerization inhibitors (*i.e.*, vinblastine and vincristine) and topoisomerases inhibitors (*i.e.*, doxorubicin and SN38). When activated, NF-κB promotes drug resistance by inducing the transcription of genes such as miRNAs, as well as genes codifying for prosurvival factors, anti-apoptotic effectors, and efflux pumps.

In addition to promote initiation and tumor progression, NF-κB signaling fosters cancer resistance to chemotherapy. Although chemotherapy is the gold standard option for many types of cancers, multi-drug resistance (MDR) occurring in late/advanced stages significantly limits its long-term efficacy. Several authors reported that various anti-cancer drugs can activate NF-κB pathway by different mechanisms (**Figure 1**). The microtubule stabilizer paclitaxel, triggers NF-κB cascade by binding the toll-like receptor 4 (TLR4) (52). Other microtubule polymerization inhibitors, such as vinblastine and vincristine, can activate NF-κB, specifically by inducing protein kinase C-mediated phosphorylation and subsequent degradation of IκBα (53, 54). Another class of drugs able to induce NF-κB are the topoisomerases inhibitors, such as doxorubicin and SN38, that act by directly activating IKK complex (55). Once activated, NF-κB promotes chemoresistance in different ways, including the induction of anti-apoptotic genes, thus increasing resistance to drug-induced damage, and the overexpression of efflux pumps to prevent xenobiotic accumulation. In A549 human lung adenocarcinoma cells, chemotherapy-induced NF-κB activation leads to the expression of anti-apoptotic proteins like BCL-xL and BFL1 that in turn promote cancer cell survival (56). Furthermore, NF-κB induces resistance to apoptosis by upregulating the expression of inhibitors of apoptosis proteins (IAPs) (57) and suppressing the TNF-related apoptosis-inducing ligand (TRAIL) pathway (58). In addition, activation of NF-κB signaling can lead to chemoresistance by directly inducing the expression of efflux pumps proteins, such as human multidrug resistance protein 1 (MDR1), also known as P-glycoprotein 1 (P-gp), and ATP-binding cassette sub-family B member 1 (ABCB1). P-gp is an ATP-dependent transporter with a broad spectrum of activity, and it is able to efflux nonionic and amphipathic xenobiotics like anthracyclines, vinca-alkaloids and taxanes. It has been observed that NF-κB can transactivate the promoter of *MDR1 (* 59) and that the inhibition of this signaling results in the downregulation of *MDR1* in different types of cancers (60–62). NF-κB is also a transcriptional regulator of cyclooxygenase-2 (COX-2) (63), whose activity showed a strong correlation with P-gp expression in hepatocellular carcinoma (64) and colorectal cancer (65), and with multidrug resistance protein 4 (MRP4) in lung cancer (66). Another important mechanism in NF-κB-mediated drug resistance involves microRNAs (miRNAs). In this regard TRAIL cascade activation induces NF-κB with subsequent upregulation of miR-21 and miR-100, which in turn activate TNF Receptor Associated Factor 7 (TRAF7) and sustain NF-κB signaling, thus generating a positive feedback loop that suppresses TRAIL-induced apoptosis (67). NF-κB is also involved in radiotherapy resistance through the transcription of prosurvival genes that mediate resistance to ionizing radiation such as DNA-damage sensor ataxia-telangiectasia mutated (ATM) and cyclin B1 (68). In addition, overexpression of several miRNAs, such as miR-125b and miR-668, leads to NF-κB activation by targeting TNF alpha induced protein 3 (TNFAIP3/A20) and IκBα, respectively, thus promoting radioresistance in nasopharyngeal carcinoma (69) and in breast cancer (70). These findings show that NF-κB activation induces drug resistance at multiple levels and that its inhibition could represent an efficient approach to improve clinical outcome.

### EVs in TME-Cancer Cell Crosstalk and Drug Resistance

#### EVs Roles in the Bidirectional Cell to Cell Communication Within the TME

**Figure 2:**
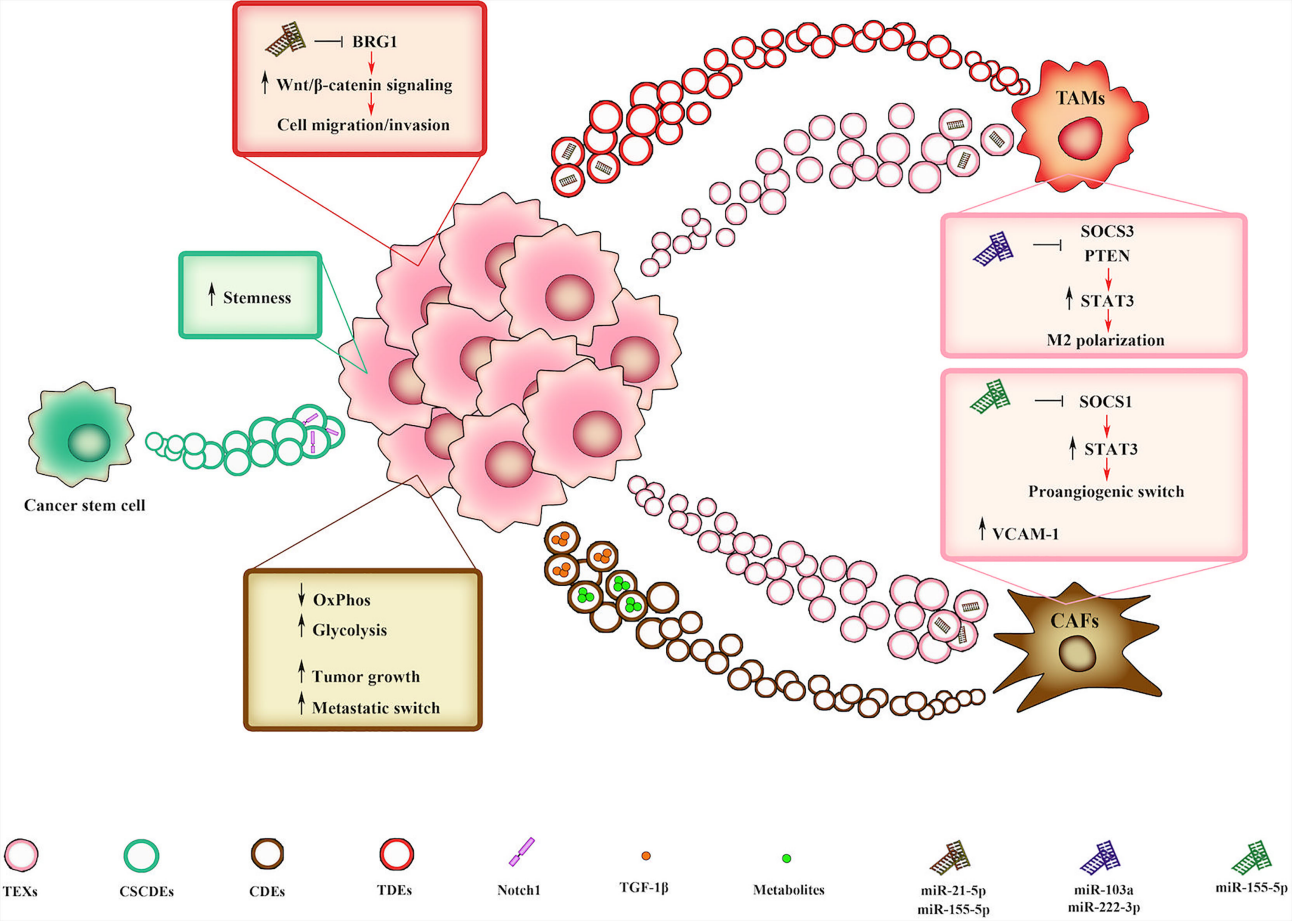
EV-mediated interplay within the TME mediates tumor progression. CAF-derived exosomes (CDEs) promote either metabolism deregulation or metastatic switch in tumor cells by delivering intact metabolites or TGF-β, respectively. TAMs-derived exosomes (TDEs) loaded with miR-21-5p and miR-155-5p can promote cell migration and invasion by downregulating BRG1 gene, thus sustaining Wnt/β-catenin signaling. CSCs-derived EVs (CSCDEs) confer stemness traits to recipient cells by delivering notch receptor 1 (notch1) protein. In turn, tumor-derived exosomes (TEXs) induce CAF transformation and macrophage M2 polarization by delivering miR-155-5p, miR-103a and miR-222-3p, that in turn affect STAT3 signaling.

The crosstalk between cancerous and surrounding cells in TME through EVs is essential to sustain tumor growth, change cell phenotype and induce metastatic switch (71) (**Figure 2**). The key role of EVs in TME is due to their capacity to transfer proteins, lipids, nucleic acids, and membrane receptors, thus providing different information based on the specific composition of their cargo. Exosome secretion and its intracellular trafficking involve a subset of small GTPase named Rabs which are demonstrated to be expressed in a context-dependent manner (72–74). In some cancers, Rabs overexpression is linked with tumor progression and worse clinical outcome (75). Once released, exosomes interact with target cells through specific receptors or molecules such as intercellular adhesion molecule 1 (ICAM1) and heat shock protein 70 (Hsp70), expressed on dendritic cell-derived and on mast cell-derived exosomes, respectively. While ICAM 1 is recognized by lymphocyte function-associated antigen 1 (LFA-1) (76, 77), Hsp70 interacts with low density lipoprotein receptor-related protein 1 (LRP1/CD91) on antigen presenting cells (78). Due to their crucial role in cell-cell communications, exosomes activity in TME has been associated with tumor progression and induction of metastatic phenotype (79, 80). Importantly, the bidirectional cell to cell communication between tumor and stromal cells sustains cancer progression toward advanced stages (81). Within TME, the communication between CAFs and TAMs with tumor cells is mediated by CAFs/TAMs derived exosomes. It was observed that CAF-derived exosomes (CDEs) containing intact metabolites are able to promote oncogenic transformation, by inhibiting oxidative phosphorylation and increasing glycolytic metabolism (82, 83). Moreover, CAFs induce tumor growth and metastatic phenotype switching by producing exosomes with high concentration of TGF-1β (84). Lan et al. demonstrated that TAMs-derived exosomes also mediate cell migration and invasion in colon cancer *via* miRNAs (miR-21-5p and miR-155-5p)-mediated downregulation of *BRG1* gene (85), whose decreased expression is responsible for Wnt/β-catenin signaling activation (86). On the other hand, tumor cells modulate and “re-educate” surrounding cells by secreting tumor-derived exosomes (TEXs). Xiao et al. showed that melanoma-derived EVs promote CAFs transformation by inducing VCAM-1 expression *via* extracellular signal-regulated kinase 1/2 (ERK1/2)-activation (87). Moreover, miR-155-5p in melanoma-derived exosomes triggers the proangiogenic switch of CAFs by targeting suppressor of cytokine signaling 1 (SOCS1), thus activating the janus kinase (JAK)2/signal transducer and activator of transcription (STAT)3 (JAK2/STAT3) pathway (88). As to TAMs transformation, Hsu and collaborators reported that in hypoxic lung cancer miR-103a-loaded EVs promoted M2 macrophage polarization by inhibiting phosphatase and tensin homolog (PTEN) and enhancing protein kinase B (PKB/Akt) and STAT3 activity (89). Moreover, ovarian cancer-derived exosomes induce macrophages polarization by delivering miR-222-3p which target SOCS3, thus sustaining STAT3 signaling (90). Furthermore, cancer stem cell (CSCs)-derived EVs (CSCDEs) play a significant role in TME remodeling. Sun et al. showed that glioblastoma stem cell (GSCs)-derived exosomes are able to confer stemness traits and enhance tumorigenicity in non-GSC glioma cells by delivering notch receptor 1 (notch1) protein and activating Notch signaling in recipient cells (91). Collectively, these studies underline the central roles of EVs in sustaining TME remodeling and fostering cancer progression.

#### EVs-Mediated Chemotherapy Resistance

**Figure 3:**
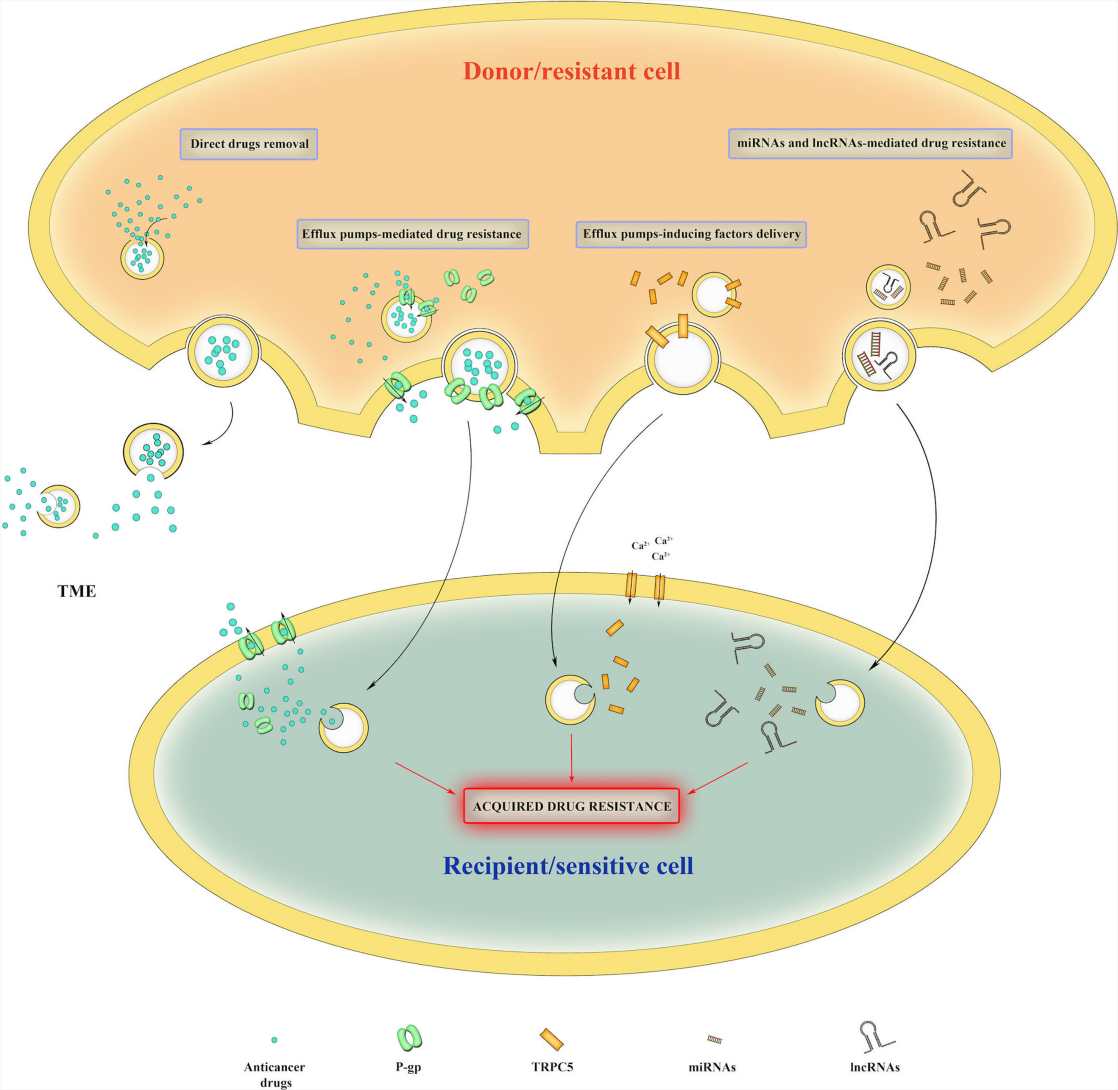
Mechanisms of EV-mediated drug resistance. EVs can directly remove intracellular drugs from cancer cells. They can also deliver efflux pumps, efflux pumps-inducing proteins, miRNAs and lncRNAs to recipient cells, thus conferring them drug resistance traits.

In addition to promote tumor growth and progression, EVs also play a pivotal role in cancer drug resistance by taking part in several processes (*i.e.*, direct removal of drugs, incorporation of efflux pumps and miRNAs/long non-coding RNAs (lncRNAs) delivery) (**Figure 3**). Direct removal of drugs from intracellular space has been reported by Shedden et colleagues; they observed the release of doxorubicin-containing vesicles in doxorubicin-treated MCF-7 breast cancer cells and demonstrated that vesicles accumulated the drug passively. Moreover, they also founded a correlation between vesicle-shedding-associated gene expression and doxorubicin resistance in several cancer cell lines (92). Furthermore, Federici and collaborators identified cisplatin-enriched exosomes in human metastatic melanoma cells and founded that EVs-mediated drug elimination is enhanced by tumor acidic microenvironment (93). EVs are also able to incorporate efflux pumps which can actively transport drugs into intraluminal space; drug resistant cancer cells can transfer efflux pumps *via* EVs to the sensitive surrounding ones, thus conferring them resistance traits (94–97). In addition, EVs can incorporate factors that induce the expression of efflux pumps. In this regard, Ma et al. demonstrated that adriamycin-resistant breast cancer cell line MCF-7/ADM showed elevated levels of Ca^2+^-permeable channel TRPC5, and that TRPC5 expression is essential for P-gp induction (98). The suppression of TRPC5 activity as well as of P-gp expression reduced drug resistance and tumor growth both *in vitro* and *in vivo* suggesting that inhibiting either P-gp or TRPC5 could be an attractive strategy to overcome drug resistance (98). They also founded that TRPC5-carrying EVs released from MCF-7/ADM cells can transfer chemoresistance properties to drug sensitive recipient cells (99). Accordingly, Wang et al. showed that exosomes-carrying Ca^2+^-permeable channel TRPC5 acts as a noninvasive chemoresistance marker that could predict chemoresistance in metastatic breast cancer patients (100). EVs can also transport drug-metabolizing enzymes, such as glutathione S-transferase P1 (GSTP1), that contribute to the intracellular detoxification as observed in chemotherapy-resistant breast cancer (101). Another crucial mechanism in drug resistance involves miRNA and lncRNA. As reported by Lunavat and collaborators, miR-211–5p, which is overexpressed in response to treatment with BRAF inhibitors (Vemurafenib and Dabrafenib), induced drug resistance in melanoma cells (102). Furthermore, Mikamori et al. evidenced that long exposure to gemcitabine increased miR-155-loaded EVs secretion in pancreatic ductal adenocarcinoma, thus conferring drug resistance through inhibition of pro-apoptotic stress-induced p53 target gene tumor protein p53-inducible nuclear protein 1 (TP53INP1) (103). Again, Shen et al. showed that breast cancer cells treated with sublethal dose of chemotherapeutic drugs released miR-9-5p, miR-195-5p, and miR-203a-3p-enriched EVs, which simultaneously targeted transcription factor One Cut Homeobox 2 (ONECUT2), thus conferring stemness and resistance traits in recipient cells (104). Qu et al. founded that, in renal cell carcinoma (RCC), lncARSR (lncRNA Activated in RCC with Sunitinib Resistance)-loaded exosomes competitively bind miR-34/miR-449 and enhance AXL and c-MET receptors signaling that are responsible for Sunitinib resistance through activation of STAT3, AKT, and ERK signaling (105). In light of this finding, lncARSR represent a potential therapeutic target to overcome sunitinib resistance in RCC (105). Other two lncRNAs involved in drug resistance are lncRNA urothelial carcinoma-associated 1 (UCA1) and lncRNA prostate androgen-regulated transcript 1 (PART1); Yang et al. founded that exosomal UCA1 is associated with Cetuximab resistance in colorectal cancer and could predict clinical outcome of Cetuximab therapy (106), while Kang and collaborators demonstrated that lncRNA PART1 is able to confer resistance to Gefitinib in esophageal squamous cell carcinoma by inducing B-cell lymphoma 2 (Bcl-2) expression through inhibition of miR-129 (107).

In summary, these findings show how EVs can exploit different mechanisms to sustain the acquisition of drug resistance by cancer cells, making them a central player in tumor evolution.

## Linking NF-κB and EVs Activity in Cancer Progression and Therapy Resistance

### Reciprocal Regulation Between EVs and NF-κB Signaling in the TME

**Figure 4:**
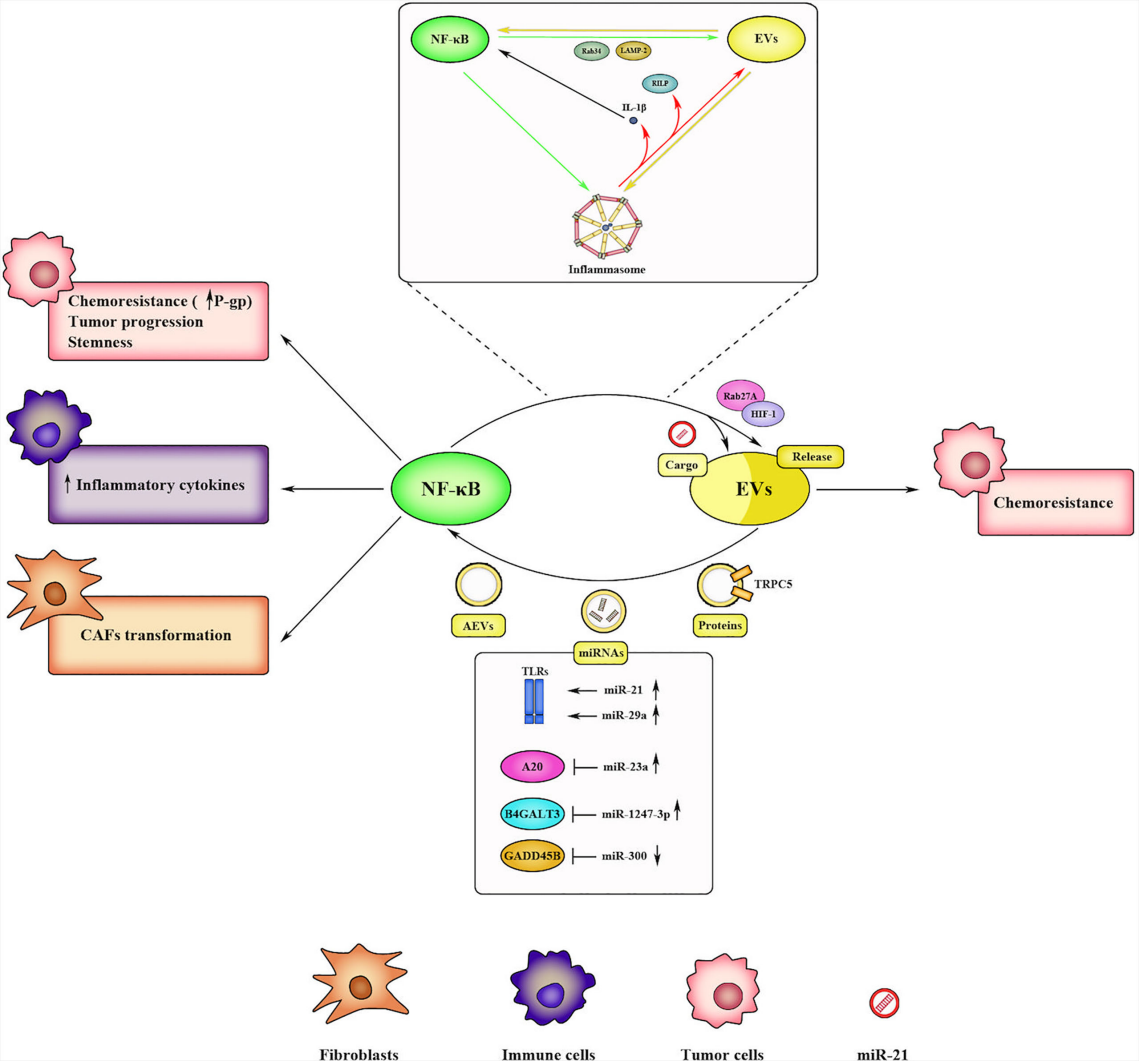
Bidirectional communication between NF-κB and EVs within the TME. NF-κB promotes EV-mediated chemoresistance in cancer cells by regulating the expression of EV-releasing factors (*i.e.*, Rab27A, Rab34, LAMP-2, HIF-1). NF-κB also affects EV cargo by inducing miRNA transcription (*i.e.*, miR-21). Reciprocally, EVs activate NF-κB through several mechanisms, such as by acting as DAMPs or delivering specific proteins and miRNAs. Upon activation, NF-κB induces chemoresistance, progression, and stemness in tumor cells, inflammatory cytokines release in immune cells, and CAFs transformation in fibroblasts. Inflammasome often mediates the crosstalk between NF-κB and EVs. Activated inflammasome promotes EV release through specific factors (*i.e.*, RILP) and sustains NF-κB signaling through IL-1β production. In turn, NF-κB regulates inflammasome activation by promoting priming step, and reciprocally, EVs induce inflammasome signaling, thus generating a positive feedback loop.

NF-κB is directly involved in EVs trafficking and EVs-mediated chemoresistance, and, at the same time, EVs are responsible for NF-κB activation (**Figure 4**).

Hypoxia is recognized as a hallmark of cancer (108) and promotes the immunosuppressive phenotype within the TME (109). In addition, hypoxia promotes EVs biogenesis and release in order to support intercellular communication and to compensate nutrient’s deficiency and oxygen starvation in cancer cells (110–112). In response to oxygen starvation, cancer cells activate hypoxia-inducible factors (HIFs) (113), a family of transcription factors involved in adaptation to hypoxic condition through modulation of angiogenesis, metastasis, and drug resistance (114), as well as in the regulation of small Rab GTPases (*i.e.*, Rab22A) which in turn control intra- and intercellular trafficking of EVs (112). It is well-known that NF-κB, in response to inflammatory stimuli, upregulates HIF-1α and HIF-1β expression (115–117). Notably, hypoxia, per sé, can induce NF-κB, leading to an inflammatory response (118). Indeed, it has been observed that inhibition of oxygen sensors prolyl hydroxylases (PHDs) by low oxygen tension, stabilizes IKKβ with subsequent p65 nuclear accumulation and signaling transduction (119). Importantly, NF-κB is also able to directly regulate Rab proteins, as observed by Feng *et al.* in colon cancer stem cells. They identified a functional NF-κB binding site in the Rab27A promoter and observed that increased p65 levels induced Rab27A expression and enhanced EVs secretion in HT29 cells (120). In turn, Rab27A can promote tumor proliferation and chemoresistance *via* NF-κB, in bladder cancer (121). Indeed, Rab27A overexpression was associated with increased phosphorylation of p65 and increased expression of the antiapoptotic gene *Bcl-2*, and conversely, pharmacological inhibition of NF-κB by BAY 11-7082 abrogated cisplatin resistance and cancer cell survival (121). NF-κB activation also influence EVs cargo. Yang *et al.* reported an altered exosomal protein profile in NF-κB knockout mice following ischemia-reperfusion (I/R) injury in skeletal muscles, suggesting that NF-κB contributes to EVs production. Specifically, in the exosomes of NF-κB knockout mice, they observed upregulation of several proteins such as protease serine 1 and glyceraldehyde-3-phosphate dehydrogenase-like isoform 1, and downregulation of other factors including apolipoprotein B, complement component C3 prepropeptide, and immunoglobulin kappa light chain variable region (122). It has been reported that NF-κB can also drive transcription of specific miRNAs including miR-21; indeed, it was observed that miR-21-carrying exosomes released by TAMs induced cisplatin resistance in gastric cancer cells modulating PTEN/PI3K/AKT signaling pathway (123).

In accordance with the reciprocal interplay between EVs and NF-κB signaling, EVs can modulate NF-κB activity in recipient cells (**Figure 4**). Bretz et al. showed that exosomes from various body fluids stimulate the NF-κB-dependent production of pro-inflammatory cytokines such as IL-1β, TNF-α, and IL-6 in monocytic cell line THP-1 *via* TLR2 and TLR4 activation (124). Gastric (125) and breast cancer (126) derived TEXs induce the expression of pro-inflammatory cytokines by macrophages *via* NF-κB, and the NF-κB activation in myeloid cells could involve TLR2 signaling (126). NF-κB pathway can be also regulated by specific EVs-delivered factors (*i.e.*, proteins, miRNAs, lncRNAs), which can directly or indirectly activate/inhibit this pathway depending on the context. As previously mentioned, exosomes-carrying Ca^2+^-permeable channel TRPC5, responsible of P-gp-induced chemoresistance, are associated with IL-6 expression and increased phosphorylation of p65 in nasal polyps (127), suggesting a possible role of NF-κB in TRPC5-mediated drug resistance. Delivered miR-21 and miR-29a, can directly trigger NF-κB-mediated inflammatory response by binding murine TLR7 and human TLR8 (128). Commonly, miRNAs modulate NF-κB pathway in an indirect manner. Fang et al. showed that miR-1247-3p-carrying TEXs promote liver cancer stemness and chemoresistance *via* inhibition of beta-1,4-galactosyltransferase 3 (B4GALT3) and activation of β1-integrin–NF-κB pathway in fibroblasts (129). Activated CAFs further induce tumor progression by secreting pro-inflammatory cytokines, including IL-6 and IL-8 (129). Furthermore, Li and colleagues observed that miR-23a-enriched exosomes activate NF-κB pathway in macrophages by targeting its negative regulator A20 (130) while Chen and collaborators founded that exosomal miR-300 controls melanoma cell progression targeting GADD45B expression, a NF-κB-induced pro-survival factor (131). MiRNAs can also be downregulated to sustain NF-κB pathway. Wang et al. demonstrated that when the usually low expressed miR-192-5p is overexpressed in TAMs-derived exosomes, it suppresses endometrial cancer progression through NF-κB inhibition (132). With respect to lncRNAs, Li et al. founded that exosomal lncRNA FMR1-AS1 (FMR1 antisense RNA 1) is associated with CSC-like phenotype by binding TLR7 in female esophageal carcinoma (133). Moreover, expression of lncRNA BORG (BMP/OP-responsive gene) in triple-negative breast cancer (TNBC) (134) and lncRNA HOTAIR (HOX antisense intergenic RNA) (135) in colorectal cancer promotes chemoresistance *via* NF-κB. NF-κB activation can be triggered also by so-called “apoptotic exosome-like vesicles” (AEVs). Recently, Park and collaborators demonstrated that AEVs, during apoptotic process, act as DAMPs and activate NF-κB pathway, thus promoting an inflammatory response (136).

### EVs and NF-κB Interplay: The Crucial Role of Inflammasome

Inflammasomes represent an important link between NF-κB and EVs activity in the TME (**Figure 4**). Inflammasomes are multimeric protein complexes that are assembled upon recognition of specific pathogen-associated molecular patterns (PAMPs) and DAMPs by PRRs (137) during innate immune response. Inflammasome consists of cytoplasmic PRRs ‘sensors’ such as nucleotide-binding domain and leucine-rich repeat receptors (NLRs), the ‘adapter’ apoptosis-associated speck-like protein containing a C-terminal caspase recruitment domain (ASC), and an ‘effector’ pro-caspase 1 (138). In this context, NF-κB plays a leading role; indeed, DAMPs are recognized by toll-like receptors (TLRs), a subset of membrane-bound PRRs which trigger NF-κB cascade and subsequent transcription of IL-1β (139). IL-1β is released as precursor protein (pro-IL-1β) and is processed in its active form by caspase-1 (140) through the formation of a multi-protein complex termed inflammasome. Although the existence of several NLRs has been reported, nucleotide-binding oligomerization domain (NOD)-like receptor (NLR) family pyrin domain containing 3 (NLRP3) is the best characterized inflammasome. Activated NF-κB upregulates NLRP3 (141, 142) that is necessary for the inflammasome assembly and for the pro-IL-1β processing. The process that lead to the inflammasome activation consist of two phases, priming and activation (143). In the priming step, NF-κB induce transcriptional upregulation of NLRP3 inflammasome components while the activation step led to full induction and inflammasome complex formation. In detail, NLRs act as DAMPs sensors leading to recruitment of ASC and subsequent activation of caspase-1 thus promoting processing of pro-IL-1β in active IL-1β. Importantly, it is known that inflammasome activation induce EVs secretion (144) by several mechanisms. One of those could be represented by *de novo* synthesis and production of IL-1β, which, in turn, activate NF-κB signaling and induce the expression of factors involved in EVs secretion. Gutierrez et al. demonstrated that activation of NF-κB can induce several membrane-trafficking regulators such as lysosome-associated membrane protein 2 (LAMP-2) and ras-related protein Rab34 (145). EVs secretion can also be promoted by inflammasome itself through caspase-1-dependent cleavage of the trafficking adaptor protein rab interacting lysosomal protein (RILP) (146). Furthermore, inflammasome-derived exosomes can activate NF-κB in macrophages leading to their pyroptosis *via* up-regulation of NLRP3 and pro-IL1β (147). While these findings show how inflammasome affects EVs secretion, it is worthy to note that EVs can also influence inflammasome activity, thus revealing a bidirectional crosstalk. Hence, EVs can either positively or negatively affect inflammasome activity depending on the nature of the EVs releasing cells (148). Exosomes from LPS-treated macrophages are able to activate NLRP3 inflammasome in AML-12 hepatocytes (149). Again, EVs from palmitate-treated Huh7 hepatocytes induce production of IL-1β in mouse bone marrow-derived macrophages (150) and exosomes from ARPE-19 exposed to photooxidative blue-light activate NLRP3 inflammasome *in vitro (* 151). However, inflammasome activation is repressed in THP1 cells treated *in vitro* with human amniotic fluid derived EVs, as well as in cardiomyocytes isolated from a mouse model of doxorubicin-induced cardiotoxicity following treatment with embryonic stem cell-derived EVs (152, 153). Although inflammasome involvement in cancer is still debated and sometimes controversial, several findings underline its role in tumorigenesis, cancer progression and drug resistance (154). In this context, NLRP3 inflammasome activity is associated with carcinogenesis in head and neck squamous cell carcinoma (155) and with proliferation and migration in A549 lung cancer cells (156).

Further, TEX-mediated inflammasome activation in non-cancer cells within the TME was shown to exacerbate inflammatory response and sustain tumor progression. In prostate cancer, EVs released from advanced-stage tumor cells were found to activate inflammasome in non-cancerous prostate cells and induce M2 polarization in THP1 (157). Moreover, Liang et al. showed that tripartite motive containing 59 (TRIM59)-loaded EVs released from lung cancer cells promoted tumor progression *in vitro* and *in vivo*. Mechanistically, TRIM59 promoted abhydrolase domain containing 5 (ABHD5) degradation and activation of NLRP3 inflammasome in macrophages, which in turn released proinflammatory cytokines, thus sustaining cancer cell proliferation and invasion (158). Hwang et al. also demonstrated that colorectal CSCs-derived exosomes induced IL-1β expression in neutrophils *via* NF-κB, thus promoting tumorigenesis in colorectal cancer cells (159). Moreover, inhibition or deletion of inflammasome components NLRP3, ASC, or caspase-1 is protective against pancreatic ductal adenocarcinoma as it is associated with the reprogramming of innate and adaptive immunity in TME (160). Inhibition of NLRP3 inflammasome also suppresses metastatic potential of melanoma cancer cells (161). Inflammasome is also involved in drug resistance and can be activated following chemotherapeutic treatments, likely as result of NF-κB stimulation. Zhai et al. founded that NLRP1 inflammasome activation induces drug resistance in melanoma cells through release of IL-1β (162). Again, Theivanthiran et al. observed activation of NLRP3 inflammasome following treatment with anti-PD-1 checkpoint inhibitor with subsequent infiltration of granulocytic myeloid-derived suppressor cells and reduction of antitumor response (163). As reported by Feng et al. inflammasome activation is also involved in in 5-fluorouracil resistance of oral squamous cell carcinoma (164).

All together, these findings highlight an intricate interplay between NF-κB and EVs activity which influence tumor growth and drug resistance. Although further studies are needed to fully elucidate the molecular mechanisms underlying this reciprocal regulation between NF-κB and EVs, it could represent yet another way through which NF-κB orchestrate tumor behavior within the TME.

## Conclusions

The bulk of evidence summarized herein shows that EVs play a key role in drug resistance through several mechanisms, including direct load and expulsion of chemotherapeutics, as well as delivery of pro-survival, anti-apoptotic, and stemness-associated factor cargo. The pharmacological target of EV biogenesis, uptake, and transfer to suppress chemoresistance has shown promise, but most of the data obtained were generated *in vitro* (165), and further *in vivo* investigation are needed. Another potential issue is represented by the heterogeneity of EVs, as not all EV communication is pro-tumorigenic. Thus, a better understanding of the molecular mechanisms underlying EV processes will significantly contribute to develop more specific approaches for overcoming drug resistance. In this review, we discuss the complex crosstalk between NF-κB pathway and EVs, as NF-κB activation affects EVs formation and release, and EVs, in turn, can trigger NF-κB activation. Since NF-κB/EVs interplay profoundly contribute to fuel aggressive disease and desensitize cancer cells to drugs, this axis could represent a promising target. However, as seen for EVs, targeting NF-κB core pathway produces off target effects due to the lack of anti-cancer specificity, as this factor is a master regulator of several biological processes. Therefore, the identification of NF-κB targets involved in this interplay in specific cancer models could be a useful strategy to overcome NF-κB/EV-mediated resistance.

## Author Contributions

MDVN and DVec wrote the manuscript. IF, DVer and MP made the figures. EA DC and FZ revised the manuscript. All authors contributed to the article and approved the submitted version.

## Funding

The work was supported by the MIUR PRIN grant n°2017WLKYAM_1 to FZ. IF is supported by Experimental Medicine PhD programme of University of L’Aquila.

## Conflict of Interest

The authors declare that the research was conducted in the absence of any commercial or financial relationships that could be construed as a potential conflict of interest.

## Publisher’s Note

All claims expressed in this article are solely those of the authors and do not necessarily represent those of their affiliated organizations, or those of the publisher, the editors and the reviewers. Any product that may be evaluated in this article, or claim that may be made by its manufacturer, is not guaranteed or endorsed by the publisher.
